# Exploring CDKN1A Upregulation Mechanisms: Insights into Cell Cycle Arrest Induced by NC2603 Curcumin Analog in MCF-7 Breast Cancer Cells

**DOI:** 10.3390/ijms25094989

**Published:** 2024-05-03

**Authors:** Felipe Garcia Nishimura, Beatriz Borsani Sampaio, Tatiana Takahasi Komoto, Wanessa Julia da Silva, Mariana Mezencio Gregório da Costa, Gabriela Inforçatti Haddad, Kamila Chagas Peronni, Adriane Feijó Evangelista, Mohammad Hossain, Jonathan R. Dimmock, Brian Bandy, Rene Oliveira Beleboni, Mozart Marins, Ana Lucia Fachin

**Affiliations:** 1 Unidade de Biotecnologia, Universidade de Ribeirão Preto (UNAERP), Ribeirao Preto 14096-900, Brazil; felipegnishi@hotmail.com (F.G.N.); beatrizborsani@gmail.com (B.B.S.); tattytk@hotmail.com (T.T.K.); wanessa.silva@sou.unaerp.edu.br (W.J.d.S.); mariana061965@hotmail.com (M.M.G.d.C.); gabrielaihaddad@gmail.com (G.I.H.); rbeleboni@unaerp.br (R.O.B.); mmarins@unaerp.br (M.M.); 2 Instituto para Pesquisa do Cancêr (IPEC), Guarapuava 85051-060, Brazil; kamila@ipec.org.br; 3 Sergio Arouca National School of Public Health, Oswaldo Cruz Foundation, Manguinhos, Rio de Janeiro 21040-900, Brazil; adriane.feijo@gmail.com; 4 School of Sciences, Indiana University Kokomo, Kokomo, IN 46904, USA; mohoss@iu.edu; 5 College of Pharmacy and Nutrition, University of Saskatchewan (USask), Saskatoon, SK S7N 5A2, Canada; jr.dimmock@usask.ca (J.R.D.); b.bandy@usask.ca (B.B.)

**Keywords:** cancer, breast cancer, curcumin, curcumin analogs, MCF-7, cell cycle arrest, p21, transcriptomics

## Abstract

Breast cancer stands out as one of the most prevalent malignancies worldwide, necessitating a nuanced understanding of its molecular underpinnings for effective treatment. Hormone receptors in breast cancer cells substantially influence treatment strategies, dictating therapeutic approaches in clinical settings, serving as a guide for drug development, and aiming to enhance treatment specificity and efficacy. Natural compounds, such as curcumin, offer a diverse array of chemical structures with promising therapeutic potential. Despite curcumin’s benefits, challenges like poor solubility and rapid metabolism have spurred the exploration of analogs. Here, we evaluated the efficacy of the curcumin analog NC2603 to induce cell cycle arrest in MCF-7 breast cancer cells and explored its molecular mechanisms. Our findings reveal potent inhibition of cell viability (IC50 = 5.6 μM) and greater specificity than doxorubicin toward MCF-7 vs. non-cancer HaCaT cells. Transcriptome analysis identified 12,055 modulated genes, most notably upregulation of *GADD45A* and downregulation of *ESR1*, implicating *CDKN1A*-mediated regulation of proliferation and cell cycle genes. We hypothesize that the curcumin analog by inducing *GADD45A* expression and repressing *ESR1*, triggers the expression of *CDKN1A*, which in turn downregulates the expression of many important genes of proliferation and the cell cycle. These insights advance our understanding of curcumin analogs’ therapeutic potential, highlighting not just their role in treatment, but also the molecular pathways involved in their activity toward breast cancer cells.

## 1. Introduction

Among the most common types of cancer, breast cancer has emerged as the foremost prevalent malignancy worldwide, ascending to the fourth position in terms of fatality rates [1,2]. The prognosis of breast cancer relies upon multifarious factors, notably the stage at diagnosis and the presence of molecular markers. These markers wield substantial influence over treatment strategies, dictating therapeutic approaches in clinical settings [3].

Normal breast cells and some breast cancer cells can be subjected to histological and molecular classification, predicated upon the presence or absence of estrogen receptors (ER), progesterone receptors (PR), human epidermal growth factor receptor 2 (HER2), and the expression levels of the cell division regulatory protein KI-67 [4,5,6]. These hormones and protein orchestrate cellular growth, thereby crucially impacting the progression and dissemination of cancer. Breast cancer cells may have one, both, or none of these receptors. The intricate interplay between these receptors delineates critical prognostic insights, with hormone receptor-positive tumors generally demonstrating a more favorable prognosis owing to their relatively slower growth kinetics and heightened susceptibility to hormone-based therapies [7]. Conversely, receptor-negative tumors, also referred to as triple-negative, exhibit a more aggressive phenotype.

Knowing a tumor’s hormone receptor status assumes importance in clinical decision-making, guiding the selection of treatment regimen. A good example lies in the case of breast cancer exhibiting HER2 overexpression, for which the target therapy is trastuzumab, a monoclonal antibody that works by binding to the HER2 receptors on the surface of cancer cells, thereby inhibiting their growth and signaling pathways [8].

Understanding the molecular mechanisms underlying hormone receptor signaling in breast cancer cells is essential for developing new treatment strategies and drugs. Researchers are continually studying these receptors to identify novel therapeutic targets and improve outcomes for breast cancer patients. And as the incidence of cancer continues to escalate annually, researchers seek to improve treatments to achieve more specificity and efficacy [7,9]. While chemotherapeutic agents targeting singular molecular entities, termed monotargeted drugs, boast commendable specificity, the specter of drug resistance looms large, underscoring the imperative of overcoming this challenge through concerted scientific endeavors [10].

Natural compounds play a vital role in the discovery and advancement of novel anticancer drugs owing to their diverse chemical structures and biological activities [11,12,13]. These compounds are screened for their biological activities against cancer cells in vitro (in cell culture) and in vivo (in animal models). Those displaying promising activity undergo further investigation to elucidate their mechanisms of action and potential as anti-cancer agents [14].

With a wide array of chemical structures, natural compounds offer a rich source of chemical diversity for drug discovery [15]. This factor enhances the probability of identifying compounds with unique mechanisms of action or enhanced efficacy compared to existing anticancer drugs.

Derived from various sources such as plants, marine organisms, fungi, and microorganisms, natural compounds have inspired the development of synthetic analogs with improved pharmacological properties [16]. For example, the discovery of vinca alkaloids from the periwinkle plant led to the development of semisynthetic analogs like vinblastine and vincristine, which are used in cancer chemotherapy [17].

Moreover, natural compounds can undergo chemical modifications to bolster their potency, selectivity, and pharmacokinetic properties [18,19]. Medicinal chemists employ techniques like structure–activity relationship (SAR) studies and combinatorial chemistry to design and synthesize analogs with enhanced anticancer activity [20,21].

A good example of a natural compound extensively studied for its health benefits and broad spectrum of biological activities is curcumin [22,23,24,25]. This compound is predominantly found in the dry matter of the rhizomes of *Curcuma longa*. While it is renowned for its culinary uses (as a key component of spicy curry), *C. longa* is also celebrated for its array of health-promoting properties, including digestive support, immune modulation, allergy prevention, antimicrobial activity, anti-inflammatory effects, wound-healing properties, and potent antioxidant action, among others [24].

Investigating the molecular mechanisms of curcumin more deeply, research has demonstrated its potent anticancer effects. Curcumin’s antiproliferative action can be attributed to its capacity to modulate protein kinases, regulate the cell cycle, and influence transcription factors such as NF-κB [26]. Additionally, it is known to interact with reactive oxygen species (ROS), further contributing to its multifaceted therapeutic potential in combating cancer [27,28].

Despite its beneficial effects, curcumin faces challenges such as poor water solubility, leading to low bioavailability and rapid metabolism [24]. However, these issues can be addressed by the utilization of analogs. These analogs have demonstrated efficacy in inhibiting proliferation, invasion, angiogenesis, and metastasis while also promoting apoptosis and autophagy, thereby holding significant promise for therapeutic interventions [29,30,31,32].

Our research group has engaged in collaborative efforts to investigate the antitumor activities of various curcumin analogs, many of which feature the 1,5-diaryl-3-oxo-1,4-pentadienyl group [33]. Recently, our focus has changed towards enhancing the efficacy of these analogs through the addition of a dichloroacetyl group placed in the molecule [34]. This series of compounds exerts cytotoxic effects through destabilizing the mitochondrial membrane potential and inducing of apoptosis and cell cycle arrest. A recent investigation demonstrated the antimigratory properties of NC2603 [35]. In that study, we employed the BT-20 triple-negative breast cancer cell line. Notably, the observed outcomes may diverge considerably from those of a hormone-responsive breast cancer cell such as the MCF-7 cell line, which is hormone-responsive. Consequently, our primary objective was to evaluate the potential of the curcumin analog NC2603 to induce cell cycle arrest on the MCF-7 breast cancer cell line. Furthermore, we aimed to delineate its underlying mechanism of action through comprehensive gene expression analysis.

## 2. Results

### 2.1. Cytotoxic Evaluation of NC2603: Dose-Response Analysis, IC50 Determination, and Comparative Efficacy against MCF-7 and HaCaT Cell Lines

**Figure 1:**
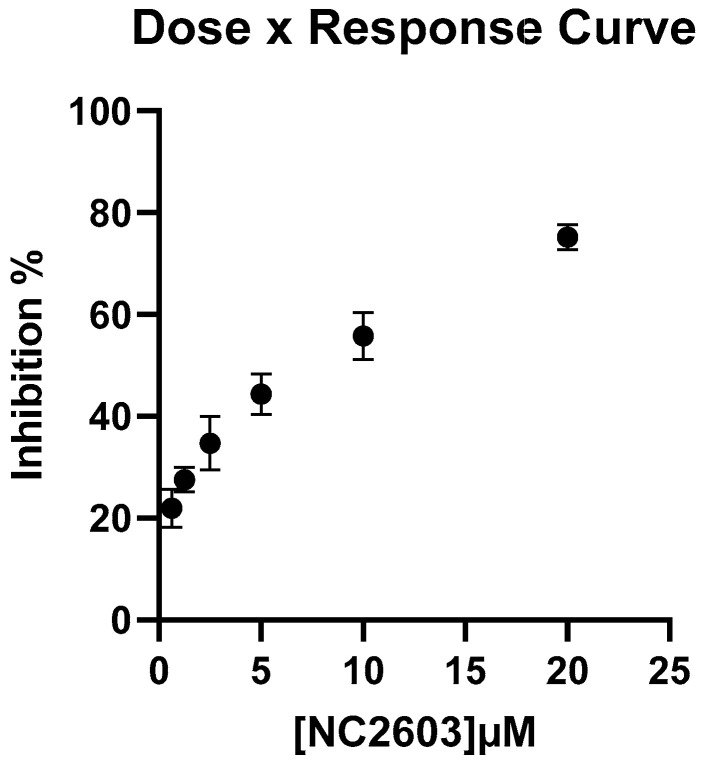
Dose–response curve of NC2603 for the MCF-7 cell line. A non-linear logarithmic curve was fitted to the data, enabling the derivation of an equation used to determine the IC50 value.

To assess the cytotoxic potential of NC2603, we constructed a comprehensive dose–response curve (Figure 1) to determine the IC50, which was determined to be 5.6 μM.

**Figure 2:**
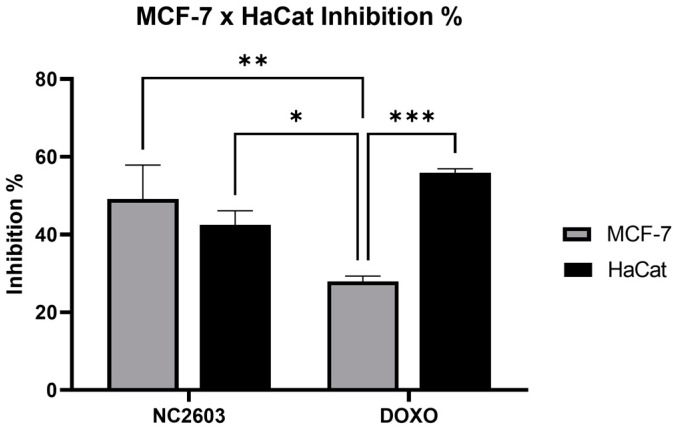
Cytotoxicity assessments of NC2603 and doxorubicin, each administered at a concentration of 5 μM, on MCF-7 and HaCaT cell lines. Treatment duration was 24 h, and cell viability was quantified using the MTT reduction assay (* *p* < 0.05, ** *p* < 0.005, *** *p* < 0.0005).

Comparative cytotoxicity assessments were conducted for analog NC2603 and doxorubicin using MCF-7 and Ha-CaT cell lines (Figure 2). Although Ha-CaT is not derived from breast tissue, it was selected as a non-tumoral cell line for comparative analysis. Despite the absence of statistically significant differences between the effects of NC2603 treatment on MCF-7 and Ha-CaT cells, significantly greater efficacy was observed against the MCF-7 cell line compared to doxorubicin treatment. Additionally, HaCaT (non-malignant) demonstrated increased susceptibility to the cytotoxic effects induced by doxorubicin treatment.

### 2.2. Differential Gene Expression Analysis: Significantly Altered Genes Regulated by Analog NC2603

**Figure 3:**
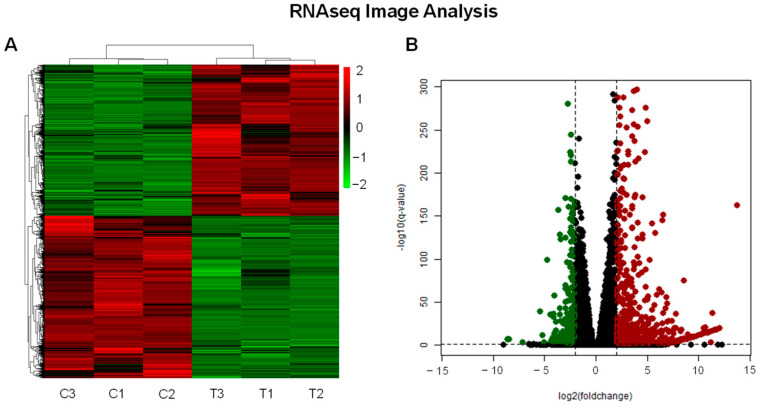
Gene expression analysis: (**A**) heatmap presenting the comprehensive differential gene expression profile of 12,055 genes following 24 h, comparing control (C) and treatment (T) conditions. (**B**) Volcano plot illustrating the differential gene expression analysis post-application of a cutoff threshold with adjusted *p*-value (PAdj) < 0.01 and log2FoldChange values *≤* −2 (green dots) and ≥2 (red dots). This refined analysis identified 872 genes exhibiting significant alterations.

The differential expression analysis conducted on the sequencing data generated a total of 12,055 differentially expressed genes (DEGs) (Figure 3A). Among these, 5806 genes were found to be repressed, while 6249 were induced. To enhance the precision of our findings, we applied a cutoff threshold, utilizing an adjusted *p*-value < 0.01 and log2FoldChange values ≤ −2 and ≥2 as parameters. This refinement process resulted in the identification of 872 DEGs (227 repressed and 645 induced) (Figure 3B). Subsequently, these genes underwent further scrutiny, focusing on their roles within the tumorigenesis process.

### 2.3. Insights through Enrichment Analysis of Differentially Expressed Genes

**Figure 4:**
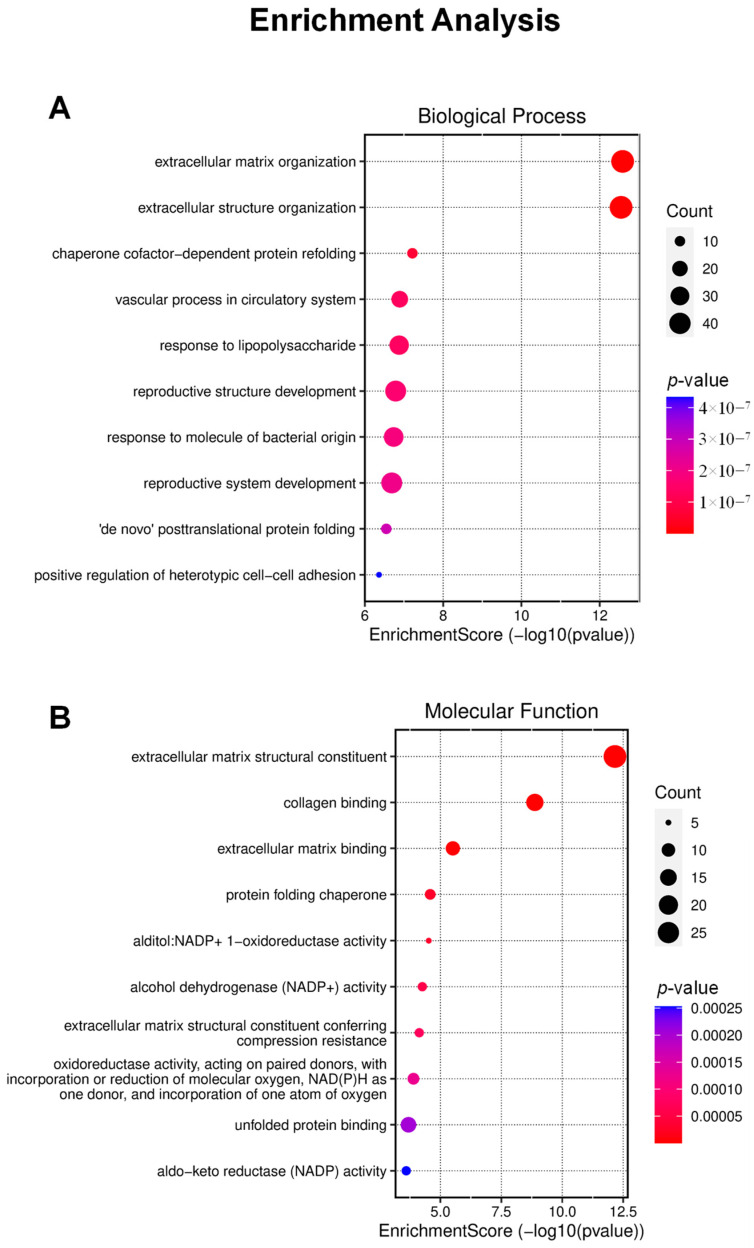
Enrichment analysis outcomes are presented, offering a visual depiction of the quantity and scoring of genes classified under biological processes (**A**) and molecular functions (**B**).

Enrichment analysis serves as a powerful tool for the identification of significantly enriched gene ontology (GO) terms among DEGs, offering valuable insights into the impacted biological processes and molecular functions (Figure 4). Nevertheless, it is important to highlight that the analysis did not yield substantial evidence concerning specific biological effects, such as alterations in the cell cycle or induction of cell death. Notably, certain sites associated with extracellular organization were identified, implying potential disturbances or alterations in cellular dynamics that could contribute to processes ultimately leading to cell demise.

### 2.4. RNAseq Data Validation

**Figure 5:**
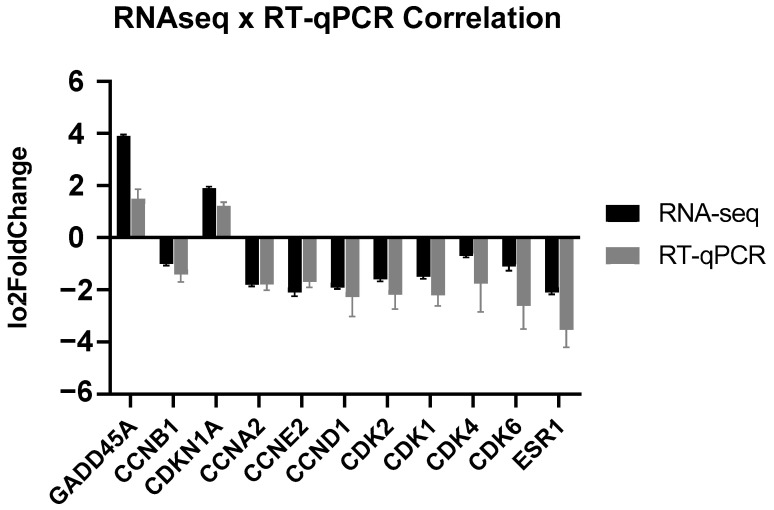
Comparison of Log2FoldChange Values from RNAseq and RT-qPCR validation experiments (Pearson correlation: R = 0.92, R squared = 0.84, *p* < 0.0001; Spearman correlation: R = 0.63, *p* < 0.05).

After treatment, two identified genes (*GADD45A* and *CDKN1A*) exhibited induction, while nine genes (*CCNB1*, *CCNA1*, *CCNE2*, *CCND1*, *CDK2*, *CDK1*, *CDK4*, *CDK6*, and *ESR1*) displayed repression. Recognizing their well-established significance in pertinent biological pathways, particularly the cell cycle, these genes were selected for further validation. Correlation analyses between RNAseq and RT-qPCR results (Figure 5) revealed a significant association (Pearson correlation: R = 0.92, R squared = 0.84, *p* < 0.0001; Spearman correlation: R = 0.63, *p* < 0.05), confirming the accuracy and reliability of our gene expression measurements utilizing both techniques.

### 2.5. Antiproliferative Effects of the Curcumin Analog NC2603

**Figure 6:**
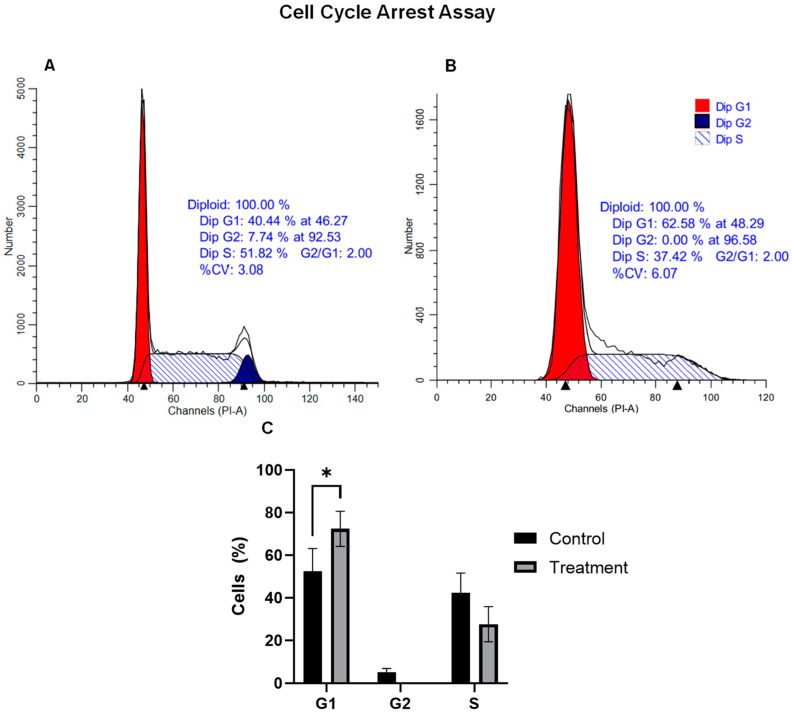
Impact of NC2603 on cell proliferation in MCF-7 Cell Line. (**A**) represents the outcomes of the control group (no treatment), (**B**) showcases the results following treatment, and (**C**) presents a graphical comparison of both datasets (* *p* < 0.03).

Based on our gene expression findings, we conducted a comprehensive assessment of cell cycle phases following NC2603 treatment. Cell cycle progression was examined by PI staining, allowing for the measurement of cell cycle distribution via flow cytometry (Figure 6).

Upon 24 h of treatment, a notable shift in the cell cycle distribution was observed compared to the control group (see Figure 6A). Specifically, the percentage of cells in the G1 phase significantly increased by 22.14% during treatment (see Figure 6B). While no statistically significant differences were identified in the S phase, this may be attributed to the absence of cells in G2 post-treatment and some presence in the control group. Consequently, our results suggest that exposure to NC2603 induces significant cell cycle arrest, specifically in the G1 phase.

### 2.6. In Silico Analysis of ESR1 and GADD45A Genes

**Figure 7:**
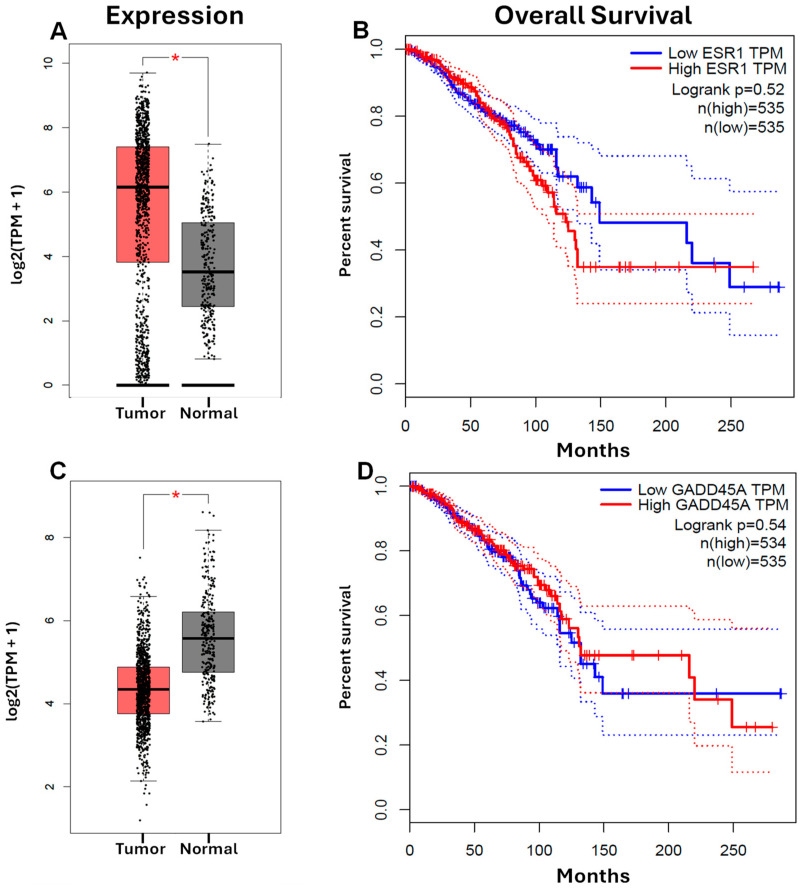
In silico analysis. (**A**) Computational analysis unveiled a notable disparity in *ESR1* expression, demonstrating elevated levels in breast cancer tissues contrasted with expression in non-tumoral tissue. (**B**) The relationship between *ESR1* expression and the overall survival of breast cancer patients was examined through the Kaplan–Meier plotter database. Dotted line represents the 95% Confidence Interval. (**C**) Analysis exhibited a contrasting pattern in *GADD45A* expression, showcasing reduced levels in breast cancer tissues juxtaposed with heightened expression in non-tumoral tissue. (**D**) Utilizing the Kaplan–Meier plotter database, we investigated the correlation between *GADD45A* expression and the overall survival of patients diagnosed with breast cancer. For both genes, *ESR1* and *GADD45A*, boxplot analysis demonstrated statistical significance with * *p* < 0.05.

The expression levels of the *GADD45A* and *ESR1* genes were compared across breast cancer and adjacent non-tumor tissues sourced from patient samples, utilizing curated data from the GEPIA database. The analysis revealed a significant upregulation of *ESR1* expression in breast cancer tissues compared to their non-tumoral counterparts (Figure 7A), whereas *GADD45A* exhibited a contrasting downregulation in tumoral tissues (Figure 7C).

To further elucidate the clinical relevance of *ESR1* and *GADD45A* expression patterns, we conducted survival analyses utilizing the Kaplan–Meier graph. Stratifying patients based on the median expression levels of both genes, we observed distinct associations with overall survival (OS). Notably, lower expression of *ESR1* correlated with a higher percentage of survival and a marginally extended survival duration (Figure 7B). Conversely, higher expression levels of *GADD45A* were associated with increased survival rates, albeit without a discernible impact on the duration of survival (Figure 7D).

## 3. Discussion

Preliminary investigations conducted by our research team and collaborators have shed light on the mechanisms and impacts of the NC2603 treatment [34,35]. Among these mechanisms, we can see the elevation of intracellular reactive oxygen species (ROS) levels, reduction in mitochondrial membrane potential, inhibition of cell proliferation, and suppression of the migration process. These findings contribute substantially to our understanding of the therapeutic potential and underlying molecular pathways influenced by NC2603 treatment. In the current study, we sought to assess the effects of NC2603 on the luminal-A (ER+, PR+, HER2−) MCF-7 breast cancer cell line. We posited that the mechanisms of action would diverge from those observed in other breast cancer subtypes, given that MCF-7 is a hormone-responsive cell line. This hypothesis is rooted in the distinct molecular characteristics and signaling pathways associated with hormone responsiveness, thus prompting a focused exploration of NC2603’s potential impact within this specific context.

Comparing the findings from the present study with research outcomes on various other cell types offers valuable insights into the mechanisms underlying the action of the curcumin analog across diverse cancer cell lines. Moreover, such comparative analyses shed light on its efficacy in addressing different subtypes of the same cancer, thus enhancing our understanding of its potential therapeutic applications. Hossain et al. [34] demonstrated that NC2603 exhibits a submicromolar IC50 against human HCT116 colon cancer cells (0.23 ± 0.054 μM). In our previous study with BT-20 cells [35], we observed an IC50 of approximately 3.5 μM for NC2603. Additionally, in MCF-7 cells, the IC50 was found to be 5.6 μM. Notably, the lowest IC50 was observed in BT-20 cells, a triple-negative breast cancer cell line. This observation could potentially be attributed to variances in the mechanisms of action across different hormonal status cell lines.

Following NC2603 treatment and subsequent RNAseq analysis, a particularly intriguing discovery emerged: the repression of the *ESR1* gene. This finding was notably absent in BT-20 cells, which do not express the ER receptor. While the implications of this observation remain speculative without corresponding protein-level experiments, it is noteworthy that modulation of certain genes and pathways associated with *ESR1* was evident.

As previously discussed, the diagnosis, prognosis, and treatment strategy for breast cancer heavily rely on the hormonal receptor status. Among breast cancers, those expressing estrogen receptors constitute the most prevalent subtype, accounting for 60–70% of cases. Consequently, hormone therapy stands as the primary therapeutic approach, with tamoxifen being the most frequently prescribed medication to block estrogen [7]. Tamoxifen binds to the estrogen receptor instead of the endogenous growth hormone 17β-estradiol, leading to an inhibition of breast cancer cell proliferation and resulting in apoptosis.

While we did not observe direct binding of NC2603 to the estrogen receptor or alteration in the protein level expression, we observed downregulation of the *ESR1* gene. We hypothesize that this downregulation could result in reduced expression of ERα, subsequently leading to decreased proliferation. Supporting this hypothesis, Mitobe et al. (2020) demonstrated that in MCF-7 breast cancer cells, knockdown of polypyrimidine tract-binding protein-associated splicing factor (PSF) significantly suppressed *ESR1* mRNA expression and ERα protein levels in a post-transcriptional manner, consequently repressing proliferation [36].

Approximately 75% of breast cancer patients clinically present estrogen receptor-positive (ER+) tumors. The regulation of ER activity and expression emerges as a pivotal area of investigation in both basic and clinical breast cancer research.

Estrogen receptors exist in two main types: the transmembrane or G-protein coupled receptor type, known as GPER, and the nuclear receptor type. The nuclear receptor type encompasses two genes, *ESR1* and *ESR2*, which encode for the corresponding protein products, ERα and ERβ. Elevated ERα activity correlates positively with breast cancer progression [37]. Understanding the intricacies of ER signaling pathways is essential for advancing therapeutic interventions and improving patient outcomes.

Liao et al. (2014) elucidated in their study the pivotal role of the estrogen receptor in driving the entry of MCF-7 cell line into the S phase of the cell cycle, thereby facilitating cellular proliferation through the upregulation of key proliferation-associated genes, namely, *PCNA* and *MKI-67*, while concurrently downregulating the expression of tumor suppressors p53 and p21 [38]. Remarkably, their investigation revealed that ERα overexpression led to the repression of p21 protein activity, resulting in a concomitant decrease in both mRNA expression and protein abundance. These profound observations underscored the intricate regulatory mechanisms orchestrated by ERα, implicating its potential role in modulating the expression of *PCNA* and *MKI-67* through the intricate regulation of p21 expression.

In this study, we observed a contrasting effect in the NC2603 treatment. Following administration, the expression of the *ESR1* gene was downregulated, whereas *CDKN1A* (p21 gene) was upregulated, alongside the downregulation of *PCNA* and *MKI67* genes. Thus, we hypothesize that one of the underlying mechanisms driving cell proliferation induced by NC2603 involves the repression of the *ESR1* gene, leading to the upregulation of *CDKN1A* and subsequently resulting in the repression of *PCNA* and *MKI67* expression.

An essential protein in this context is the p21, also known as Cip1 or Waf1, which is encoded by the *CDKN1A* gene. p21 is widely recognized as a universal inhibitor of cyclin-dependent kinases (CDKs) [39]. The cell cycle is tightly regulated by the interaction between cyclins and CDKs. The diverse functionalities of this process stem from the formation of complexes between these molecules. Specific cyclins, such as cyclin D, establish complexes with CDKs 4 and 6, while cyclins E and A form complexes with CDK2, and cyclin B associates with CDK1 [40,41].

The formed complexes play a crucial role in phosphorylating proteins belonging to the retinoblastoma family (Rb proteins). These proteins, in turn, interact with the E2F protein, exerting a critical role in regulating the expression of genes that control the cell cycle [42]. Hyperphosphorylation of Rb proteins prevents their interaction with E2F proteins, while hypophosphorylation allows this interaction. When Rb-E2F complex formation occurs, it represses the expression of genes necessary for cell cycle progression [39].

Since p21 inhibits CDKs, it plays a direct role in the formation of the Rb-E2F complex, thus serving as a crucial component in cell cycle control.

Based on the gene expression results, treatment with the NC2603 analog exerts a notable influence on the cell cycle control pathway. Induction of the *CDKN1A* gene was observed following treatment, whereas genes encoding cyclins A, B, D, and E, along with those encoding CDKs 1, 2, 4, and 6, were uniformly suppressed.

**Figure 8:**
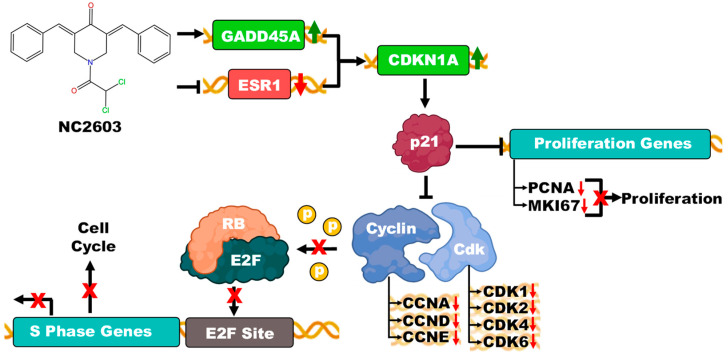
Representative scheme of the potential mechanism of action of the NC2603 analog on cell cycle arrest. The analog, while inducing the expression of the *GADD45A* gene, simultaneously repressed the expression of the *ESR1* gene (upward green arrow indicating upregulation and downward red arrow indicating downregulation). Both, under these conditions, induced the expression of *CDKN1A*. The protein p21, encoded by the *CDKN1A* gene, inhibits the formation of cyclin/CDK complexes, the majority of which had their expression repressed after treatment with the analog. Since there is no formation of cyclin/CDK complexes, the Rb protein is hypophosphorylated, thereby complexing with the E2F protein. When the Rb/E2F complex is formed, it negatively regulates the cell cycle, i.e., causing cell cycle arrest. On the other hand, the p21 protein still inhibits the *PCNA* and *MKI67* genes, which induce cell proliferation. Since treatment with the analog repressed the expression of these genes, this also becomes a possible mechanism of action of the analog. (Arrows with an ‘X’ indicate inhibition of the process).

From a protein perspective, the increased production of the p21 protein results in reduced formation of complexes between cyclins and CDKs. Consequently, this cascade leads to the hypophosphorylation of the Rb protein, facilitating its interaction with E2F and potentially culminating in cell cycle arrest, highlighting the intricate molecular interplay influenced by NC2603 analog treatment (Figure 8).

However, an alternative scenario suggests the activation of *CDKN1A* via the upregulation of *GADD45A* expression, which was also observed following NC2603 treatment. The growth arrest and DNA damage-inducible (GADD)45 proteins, are swiftly induced in response to DNA-damaging agents like UV radiation and X-ray irradiation, peaking in the G1 phase and declining during S phase. Their expression is intricately regulated at transcriptional, post-transcriptional, and post-translational levels. GADD45 proteins are integral to various cellular processes linked with stress signaling and response to cell injury, including oncogenic stress, terminal differentiation, and apoptotic cytokines, thereby influencing tumor formation. They interact with key proteins involved in DNA replication and repair, such as *PCNA* and *CDKN1A*, and directly inhibit CDK1 [43].

Some authors have demonstrated that *GADD45A* upregulation directly influences cell cycle arrest and concomitant *CDKN1A* upregulation, with implications for *TP53* functionality [44,45]. Ferraros et al. (2011) have demonstrated that some complex phenols naturally present in extra virgin olive oil phenolic extract (EVOO PE) trigger the activation of *GADD45* genes, thereby instigating the upregulation of genes associated with cell cycle arrest and apoptosis, notably including *CDKN1A* [46]. Moreover, Saha et al. have shown similar effects in a human adenocarcinoma cell line in a *TP53*-independent manner, induced by curcumin [47]. Notably, in a study utilizing phytoestrogens (phenolic compounds derived from soybean, tofu, vegetables, fruits, leaves, and grains that can bind to the ER), the treatment induced cell cycle arrest and apoptosis in a p53-dependent manner for MCF-10A (a normal breast cell line wild-type p53) and in a p53-independent manner in MDA-MB-231 (breast cancer cell line mutant p53) [48]. Both are ER- cell lines and had upregulated p21. Here, with MCF-7, an ER+ cell line wild-type p53, the results indicate that most of the processes caused by NC2603 treatment occurred independently of p53, which had its gene expression reduced (log2FoldChange = −1, PAdj = 1.6 × 10^−57^).

G1 arrest can be induced by various agents independent of *TP53* status and is often accompanied by the induction of *CDKN1A* [49,50]. Both *GADD45A* and *CDKN1A* exhibit versatile functions, mediating cell cycle arrest in both *TP53*-dependent and -independent manners and impacting phases ranging from G1/S transition to G2/M arrest [51,52,53].

Moreover, the findings from our in silico analysis offer compelling insights suggesting that treatment with the NC2603 analog holds promise for enhancing prognostic outcomes. Specifically, our investigation revealed a notable upregulation of the *ESR1* gene in tumor tissue, correlating with unfavorable prognoses, as depicted in the OS graph. Conversely, expression of *ESR1* in non-tumor tissues was associated with more favorable prognostic indicators, a trend which was further accentuated following treatment with the NC2603 analog. Similarly, the *GADD45A* gene exhibited a comparable pattern, where heightened expression levels were linked with improved prognostic outcomes, which were notably enhanced post-treatment with the analog. Consequently, irrespective of the analog’s primary mechanism of action, our findings suggest a potential indirect association with improved prognostic outcomes.

## 4. Materials and Methods

### 4.1. Materials

The curcumin analog NC2603 (shown in Figure 7) was synthesized by collaborators from the University of Saskatchewan, Canada [34]. Some reagents essential for our experimental assays, such as thiazolyl blue tetrazolium bromide (MTT), dimethyl sulfoxide (DMSO), RPMI-1640 medium, curcumin, and trypsin, were purchased from Sigma-Aldrich (St. Louis, MO, USA).

Sequencing analyses were performed utilizing Illumina technology, specifically employing the NovaSeq 6000 SP Reagent Kit, Illumina^®^ Stranded mRNA Prep, Ligation and IDT^®^ for Illumina^®^ RNA UD Indexes Set A, Li-gation (San Diego, CA, USA). For RT-qPCR experiments, we utilized the highly sensitive SYBR^®^ Green JumpStart™ Taq Ready-Mix™ and primers, also sourced from Sigma-Aldrich (St. Louis, MO, USA), ensuring accuracy and reproducibility in our gene expression analyses.

### 4.2. Cell Culture

The breast cancer cell line MCF-7 (Banco de Células do Rio de Janeiro—BCRJ, a Luminal A cell line (ER+, PR+, HER2−) and the non-tumoral human keratinocyte cell line HaCat (Cell Lines Service GmbH, Eppelheim, Germany) were both cultured in RPMI medium supplemented with 10% fetal bovine serum and maintained at 37 °C in a humidified atmosphere containing 5% CO_2_. Then, 100 U/mL of the antibiotics penicillin, streptomycin, and kanamycin were added to the culture medium [54].

### 4.3. Cell Viability

The cytotoxicity evaluation of NC2603 was conducted using the MTT assay, following the protocol proposed by Komoto et al. with modifications [55]. Cells were seeded at a density of 2 × 10^4^ cells per well in 96-well plates and allowed to adhere for 24 h. Following incubation, cells were exposed to varying concentrations of the NC2603 analog for 24 h. Doxorubicin served as a positive control, while DMSO was used as a solvent control. For comparing the effects in MCF-7 and HaCaT cells, a concentration of 5 μM was used, and for the dose–response curve construction, 20, 10, 5, 2.5, 1.25, 0.625 μM were used. Subsequently, 20 μL of MTT solution (5 mg/mL in Hanks buffer) was added to each well and incubated for an additional 3 h. The resultant formazan crystals were solubilized using 150 μL of dimethyl sulfoxide for 1 h. Absorbance was measured at 550 nm using a microplate reader (MultiSkan FC, Thermo Fisher, Waltham, MA, USA).

### 4.4. Next Generation Sequencing

First, total RNA was extracted from MCF-7 cells treated with NC2603 [5.6 μM] and control MCF-7 cells treated with DMSO [<0.2%] utilizing the RNeasy Mini Kit (Qiagen, Hilden, Germany) as per the manufacturer’s instructions. The integrity of RNA samples was thoroughly assessed using the TapeStation system (Agilent, Santa Clara, CA, USA), ensuring that only samples with RNA integrity values > 8 were included in subsequent analyses. High-quality cDNA libraries were generated using the High-Capacity cDNA Reverse Transcription Kit (Applied Biosystems, Foster City, CA, USA).

Subsequently, the samples underwent sequencing using the advanced NovaSeq 6000 system (Illumina, San Diego, CA, USA), following standardized protocols and employing the Illumina^®^ Stranded mRNA Prep kits, IDT^®^ for Illumina^®^ RNA UD Indexes Set A, and NovaSeq 6000 SP Reagent Kit.

The acquired sequencing data underwent quality control measures utilizing FastQC 0.11.9 (q < 28) and subsequent trimming with Trim Galore 0.6.7 to eliminate low-quality reads. Alignment to the hg19 reference genome (GRCh37) was performed using the STAR 2.7.9a tool (Spliced Transcripts Alignment to a Reference), ensuring accurate mapping.

Furthermore, transcript quantification was conducted employing the cutting-edge Salmon 1.5.2 tool. Differential expression analysis was executed, and differentially expressed genes (DEGs) were discerned utilizing the robust statistical methods embedded within the DESeq2 1.31.16 and EdgeR 3.38.0 software packages, facilitating comprehensive exploration of gene expression alterations induced by NC2603 treatment in MCF-7 cells.

### 4.5. RT-qPCR

A panel comprising 13 genes (*GADD45A*, *CCNB1*, *CDKN1A*, *CCNA2*, *CCNE2*, *CCND1*, *CCND2*, *CDK2*, *CDK1*, *CDK4*, *CDK6*, *PCNA*, and *ESR1*) was curated based on their involvement in relevant pathways (Table 1). This selection aimed to corroborate and complement the findings from sequencing analyses, elucidating the dynamics of gene expression in MCF-7 cells upon exposure to NC2603, utilizing the previously determined IC_50_ concentration. The expression levels were assessed via RT-qPCR methodology employing SYBR^®^ Green JumpStart™ Taq ReadyMix™ (Sigma-Aldrich, St. Louis, MO, USA) and conducted on Mx3300 qPCR System (Stratagene, San Diego, CA, USA). Each primer set was validated through three independent experiments, ensuring the reliability and reproducibility of the obtained results.

### 4.6. Enrichment Analysis

To elucidate the biological processes and molecular functions linked with the differentially expressed genes (DEGs), we performed a comprehensive enrichment analysis of gene ontology (GO) utilizing the powerful R programming language. Enrichment analysis maps were obtained from a reputable online data analysis platform (http://www.bioinformatics.com.cn/, accessed on 29 June 2023) to enhance the visualization and interpretation of enriched biological processes. This approach provides valuable insights into the underlying molecular mechanisms and functional pathways influenced by the differential gene expression.

### 4.7. Cell Cycle Progression Assay

The cell cycle analysis was performed utilizing the flow cytometry technique with propidium iodide as the staining marker. Initially, MCF-7 cells were subcultured at a concentration of 2 × 10^5^ cells/mL in 25 cm^2^ cell culture flasks, each containing 5 mL of the cell suspension (a total of 1 × 10^6^ cells). These cells were subjected to treatments with the compound NC2603 (5.6 μM) and the solvent control DMSO (<0.01%) for a 24 h period. Following treatment, supernatants were collected in 15 mL centrifuge tubes. Subsequently, cells were detached using trypsin and collected in the respective tubes along with the supernatant. The tubes were then centrifuged at 1741 G for 5 min at 4 °C.

For cell cycle evaluation, we employed the BD Cycletest™ Plus kit (BD Pharmigen™, San Diego, CA, USA), strictly adhering to the manufacturer’s recommended procedures [56]. Flow cytometry analysis was conducted utilizing a cytometer (LSR Fortessa flow cytometer (BD Biosciences, San Diego, CA, USA). Data acquisition and analysis were conducted using BD FACS Diva software (version 8.0.1).

### 4.8. In Silico Analysis

The in silico analysis for gene expression in tumoral tissues and the corresponding overall survival associated with these genes was conducted using the online tool Gene Expression Profiling and Interactive Analysis (GEPIA) (accessible at: http://gepia.cancer-pku.cn/index.html, accessed on 22 March 2024). This powerful platform facilitated comprehensive exploration of gene expression patterns and their clinical relevance, offering robust insights into the molecular landscape of cancer and its impact on patient outcomes [57].

For both boxplot and survival tests, datasets of breast invasive carcinoma (BRCA) were utilized, sourced from The Cancer Genome Atlas Program (TCGA) and The Genotype-Tissue Expression (GTEx) database. Differential analysis was conducted using one-way ANOVA with a Log2FC cutoff of 1 and a *p*-value cutoff of 0.01.

### 4.9. Statistical Analysis

For viability and cell cycle assay data analysis, we employed ordinary two-way ANOVA followed by Bonferroni’s multiple comparisons test to evaluate statistical significance. Additionally, to assess the correlation between the two datasets, we conducted both parametric (Pearson correlation) and nonparametric (Spearman correlation) tests. This comprehensive approach ensures statistical validation and provides deeper insights into the relationships between experimental variables, enhancing the reliability and interpretability of our findings.

## 5. Conclusions

This study aimed to investigate the potential of the curcumin analog NC2603 as an inducer of cell cycle arrest. Our results revealed that NC2603 effectively induced cell cycle arrest, with transcript sequencing indicating modulation of several genes crucial for cell cycle regulation. Specifically, our findings suggest that the mechanism of action of NC2603 involves the potential induction of either *ESR1* or *GADD45A*, culminating in the subsequent upregulation of *CDKN1A*, the gene responsible for encoding the p21 protein, a known regulator of the cell cycle. Another plausible scenario is that both *ESR1* and *GADD45A* are interconnected within a single pathway, collectively contributing to the modulation of *CDKN1A* expression and subsequently influencing cell cycle regulation.

Augmenting our understanding of NC2603’s mechanisms would be best achieved through the assessment of protein expression, which offers a more robust and reliable insight into its mode of action. Gene deletion techniques for *ESR1* and *GADD45A*, could elucidate the precise mechanisms underlying the effects of these genes and NC2603, enriching our understanding of their interactions and therapeutic potential.

This study advances our comprehension of the intricate molecular pathways governing *CDKN1A* expression, shedding light on the potential of NC2603 as a promising adjuvant treatment candidate. Furthermore, our findings highlight *GADD45A* and *ESR1* genes and their associated mechanisms as promising targets for therapeutic interventions or as potential biomarkers. Further investigations are warranted to fully elucidate the mechanisms underlying the effects of these genes and NC2603 on cell cycle arrest. These findings provide valuable insights for the development of targeted therapies and lay the groundwork for future research in this field.

## Figures and Tables

**Table 1:** Primer sequences for RT-qPCR.

Gene	Primer	
	Forward 5′–3′	Reverse 5′–3′
*GADD45A*	TGCGAGAACGACATCAACAT	TCCCGGCAAAAACAAATAAG
*CCNB1*	AATAAGGCGAAGATCAACATGGC	TTTGTTACCAATGTCCCCAAGAG
*CDKN1A*	GACACCACTGGAGGGTGACT	CAGGTCCACATGGTCTTCCT
*CCNA2*	CACTCTACACAGTCACGGGA	AGTGTCTCTGGTGGGTTGAG
*CCNE2*	CTTACGTCACTGATGGTGCTTGC	CTTGGAGAAAGAGATTTAGCCAGG
*CCND1*	TCTACACCGACAACTCCATCCG	TCTGGCATTTTGGAGAGGAAGTG
*CCND2*	GTTCCTGGCCTCCAAACTCA	CTTGATGGAGTTGTCGGTGTAAAT
*CDK2*	ATGGATGCCTCTGCTCTCACTG	CCCGATGAGAATGGCAGAAAGC
*CDK1*	TTTTCAGAGCTTTGGGCACT	CCATTTTGCCAGAAATTCGT
*CDK4*	CCATCAGCACAGTTCGTGAGGT	TCAGTTCGGGATGTGGCACAGA
*CDK6*	GGATAAAGTTCCAGAGCCTGGAG	GCGATGCACTACTCGGTGTGAA
*PCNA*	ATTAAACGGTTGCAGGCGTAG	ACATCTGCAGACATACTGAGTG
*ESR1*	GCTTACTGACCAACCTGGCAGA	GGATCTCTAGCCAGGCACATTC

## Data Availability

The data are available upon request from the corresponding author.
